# A New Score Unveils a High Prevalence of Mild Cognitive Impairment in Patients with Nonalcoholic Fatty Liver Disease

**DOI:** 10.3390/jcm10132806

**Published:** 2021-06-25

**Authors:** Carla Giménez-Garzó, Alessandra Fiorillo, María-Pilar Ballester-Ferré, Juan-José Gallego, Franc Casanova-Ferrer, Amparo Urios, Salvador Benlloch, David Martí-Aguado, Teresa San-Miguel, Joan Tosca, María-Pilar Ríos, Cristina Montón, Lucía Durbán, Desamparados Escudero-García, Luis Aparicio, Vicente Felipo, Carmina Montoliu

**Affiliations:** 1 Laboratory of Neurobiology, Centro Investigación Príncipe Felipe, 46012 Valencia, Spain; cgimenez@cipf.es (C.G.-G.); aurios@incliva.es (A.U.); 2 Fundación Investigación Hospital Clínico, Instituto de Investigación Sanitaria, INCLIVA, 46010 Valencia, Spain; afiorillo@incliva.es (A.F.); mapibafe@gmail.com (M.-P.B.-F.); jjgallego@incliva.es (J.-J.G.); fcasanova@incliva.es (F.C.-F.); david_marti_aguado@hotmail.com (D.M.-A.); joantosca@gmail.com (J.T.); monton_cri@gva.es (C.M.); m.desamparados.escudero@uv.es (D.E.-G.); carmina.montoliu@uv.es (C.M.); 3 Servicio de Medicina Digestiva, Hospital Clínico Valencia, 46010 Valencia, Spain; 4 Servicio de Digestivo, Hospital Arnau de Vilanova, 46015 Valencia, Spain; drbenlloch@yahoo.es (S.B.); mriosp73@hotmail.com (M.-P.R.); durban_luc@gva.es (L.D.); 5 CIBERehd, Instituto de Salud Carlos III, 28029 Madrid, Spain; 6 Departamento de Patología, Universidad Valencia, 46010 Valencia, Spain; Teresa.Miguel@uv.es; 7 Departamento de Medicina, Universidad Valencia, 46010 Valencia, Spain; 8 Departamento de Anatomía y Embriología, Universidad Valencia, 46010 Valencia, Spain; luis.aparicio@uv.es

**Keywords:** psychometric score, NAFLD, NAFL, NASH, neurological impairment

## Abstract

Patients with nonalcoholic fatty liver disease (NAFLD) may show mild cognitive impairment (MCI). The neurological functions affected remain unclear. The aims were to: (1) Characterize the neuropsychological alterations in NAFLD patients; (2) assess the prevalence of impairment of neurological functions evaluated; (3) develop a new score for sensitive and rapid MCI detection in NAFLD; (4) assess differences in MCI features between patients with nonalcoholic fatty liver (NAFL) and nonalcoholic steatohepatitis (NASH); and (5) compare neuropsychological alterations in NAFLD patients with cirrhotic patients with MCI. Fifty-nine NAFLD patients and 53 controls performed psychometric tests assessing different neurological functions: PHES (Psychometric Hepatic Encephalopathy Score) battery, d2, Stroop, Oral SDMT (Symbol Digit Modalities Test), Digit Span, number-letter test, and bimanual and visual-motor coordination tests. NAFLD patients show impairment in attention, mental concentration, psychomotor speed, cognitive flexibility, inhibitory mental control, and working memory. We developed a new, rapid, and sensitive score based on the most affected parameters in NAFLD patients, unveiling that 32% of NAFLD show MCI. Prevalence was similar in NAFL (36%) or NASH (27%) patients, but lower in NAFLD than in cirrhosis (65%). MCI prevalence is significant in NAFLD patients. Psychometric testing is warranted in these patients to unveil MCI and take appropriate measures to reverse and prevent its progression.

## 1. Introduction

Nonalcoholic fatty liver disease (NAFLD) is the primary cause of liver disease and is characterized by hepatic steatosis not attributable to the consumption of alcohol, long-term use of steatogenic medication, or monogenic hereditary disorders. The most common risk factors include obesity, insulin resistance, diabetes, and metabolic syndrome [1,2]. NAFLD prevalence is increasing as a consequence of obesity, although many patients are lean. Its prevalence in the general population is estimated at 24%, the highest rates are reported from South America and the Middle East, followed by Asia, the USA, and Europe [3], and incidence is expected to continue rising in parallel with obesity, diabetes, and other lifestyle-related diseases [4].

NAFLD can be classified as nonalcoholic fatty liver (NAFL) or nonalcoholic steatohepatitis (NASH). NAFL is defined as the presence of ≥5% hepatic steatosis without hepatocellular injury. NASH is defined as the presence of ≥5% hepatic steatosis and inflammation with hepatocyte injury (e.g., ballooning), with or without fibrosis [1], and has an estimated prevalence of around 3–6%.

Progression of chronic liver disease to cirrhosis is associated with appearance of complications such as varices, hepatic encephalopathy, hepatopulmonary syndrome, or coagulation disorders. These comorbidities impair life quality and decrease survival [5]. An early complication in cirrhotic patients is minimal hepatic encephalopathy (MHE), with mild cognitive impairment, attention deficits, psychomotor slowing, and impaired coordination [6,7,8,9]. MHE reduces the ability to perform daily life tasks and is associated with altered postural control, increased risk of falls, fractures, and hospitalizations, and of progression to overt hepatic encephalopathy, increased costs, and reduced life quality and span [6,10,11,12]. Early MHE detection may help to reverse or halt progression of MHE [13].

Patients with chronic liver disease may develop mild cognitive and/or motor impairment before reaching cirrhosis, as recently extensively reviewed [14]. Patients with NASH and obesity show mild cognitive impairment (MCI) if their levels of hyperammonemia and inflammation are sufficiently high [15]. Individuals with T2 diabetes mellitus (T2DM) or with NAFLD and T2DM have reduced cognitive performance, and preventive strategies to optimize T2DM management in patients with NAFLD are necessary [16,17]. However, this MCI and the neurological functions affected are understudied areas. It is also unclear whether these alterations are similar or different to those of cirrhotic patients with MHE. In this study, we sought to characterize neuropsychological alterations in depth, performing a wide battery of psychometric tests to assess different neuropsychological functions in NAFLD patients without obesity and the prevalence of impairment in different types of neurological functions. We then sought to develop a new score to detect MCI sensitively and rapidly in patients with NAFLD, assess any differences in profile or prevalence of MCI between patients with NAFL and NASH, and evaluate whether the neurological functions affected in NAFLD patients are similar to those in cirrhotic patients with MHE.

The Psychometric Hepatic Encephalopathy Score (PHES) is the gold standard for MHE diagnosis in cirrhotic patients [18,19]. However, different groups have shown recently that the PHES is not sensitive enough to detect all patients with mild cognitive or motor impairment, finding that a good number of cirrhotic patients classified as non-MHE by PHES show impaired performance in psychometric tests assessing certain, more specific, neurological functions [9,20,21]. More than 40% of patients classified as without MHE by the PHES test showed alterations in attention and coordination, which had passed undetected by PHES. Oral Symbol Modalities Test (SDMT), d2, and bimanual and visual-motor coordination tests were more sensitive than PHES in detecting these early changes [21]. To characterize MCI, NAFLD patients and controls were administered the PHES battery as well as an additional battery of psychometric tests evaluating other functions [21].

## 2. Materials and Methods

### 2.1. Patients and Controls

Fifty-nine patients with NAFLD were consecutively recruited from the outpatient clinics in the University Clinic and Arnau de Vilanova hospitals in Valencia, Spain. NAFLD diagnosis was based on clinical, biochemical, and ultrasonographic data. Exclusion criteria were recent (<6 months) alcohol intake, infection, recent (<6 weeks) antibiotic use or gastrointestinal bleeding, recent (<6 weeks) use of drugs affecting cognitive function, hepatocellular carcinoma, and neurological or psychiatric disorder. Patients with insulin-dependent diabetes were excluded. Fifty-three healthy volunteers without liver disease were also included. All participants were enrolled after signing written informed consent. Study protocols were in accordance with the ethical guidelines of the Declaration of Helsinki and were approved by the Research Ethics Committees of both hospitals.

After performing the psychometric tests, patients were classified as without or with MCI as described below. The characteristics of the groups are shown in Table 1.

### 2.2. Classification of NAFLD Patients as NAFL or NASH

Liver biopsies were available for clinical reasons in 46 of the 59 NAFLD patients, who were classified as NAFL or NASH as described by Kleiner et al., [22] taking into account steatosis, inflammation, and ballooning. Biopsy extraction was not clinically justified in the remaining 13 patients, who were classified according to the FAST (FibroScan-AST) score [23].

### 2.3. Neuropsychological Assessment

First, patients performed the PHES battery, the gold standard of MHE diagnosis in cirrhotic patients [18,19]. As some patients show deficits in neurological functions not fully assessed by the PHES but sensitively evaluated by psychometric tests such as Oral SDMT, d2, digit span, and visual-motor or bimanual coordination [21], patients and controls also performed these tests, as described below.

#### 2.3.1. PHES

PHES comprises five psychometric tests: Digit symbol test (DST), number connection test A (NCT-A), number connection test B (NCT-B), serial dotting test (SD), and line tracing test (LTT) [18]. The PHES score was calculated adjusting for age and education level using Spanish normality tables (http://www.redeh.org/TEST_phes.htm accessed on 25 June 2021). Patients were considered to have MHE when the score was ≤−4 points [18].

#### 2.3.2. Stroop Test

Selective attention, psychomotor speed, cognitive flexibility, and inhibitory mental control were assessed with a color-word version of the Stroop test, performing the congruent, neutral, and incongruent tasks sequentially, 45 s per task, as described in Felipo et al. [9]. The number of items correctly named was adjusted for age according to Spanish normality tables.

#### 2.3.3. Bimanual and Visual-Motor Coordination Tests

Bimanual and visual-motor coordination tests were performed as in [9] and time in minutes was recorded for each test.

#### 2.3.4. Symbol Digit Modalities Test (Oral SDMT)

This test assesses selective attention and processing speed and consists of a series of nine symbols, each one paired with a single digit labeled 1–9. The test page presents a sequence of symbols, and patients have to identify the right number corresponding to each symbol over a 90 s period [21]. The number of total items, correct pairings, and errors are registered.

#### 2.3.5. D2 Test

This test evaluates selective/sustained attention and mental concentration and provides scores reflecting three components of attentional behavior: Speed or amount of work done in a given time; accuracy of the work, and relationship between speed and accuracy [24,25]. The d2 test was performed as in [21] and uses the following parameters: Total number of characters processed (TR); right answers (RA) (number of characters correctly cancelled), omission errors (O) (number of target symbols not cancelled); commission errors (C) (number of non-target symbols cancelled); total errors (O_C) (sum of omission and commission errors); total correctly processed (TOT) (total characters processed minus total errors made); (CON) concentration performance (number of correctly minus incorrectly cancelled items).

#### 2.3.6. Digit Span

This test pertaining to the Wechsler Adult Intelligence Scale (WAIS) [26] consists of two parts: ‘digits forward’, which evaluates verbal attention and ‘digits backward’ evaluating immediate and working memory, which were performed as in [21].

#### 2.3.7. Letter–Number Sequencing or Number-Letter Test

Letter–number sequencing or the number-letter test was performed as in [21] and measures working memory, inducing a higher working memory load than digit span tasks.

### 2.4. Procedure to Classify NAFLD Patients as with or without Mild Cognitive Impairment (MCI/NMCI)

The PHES battery diagnosed only 7 out of 59 patients (12%) as having MHE. However, we observed that they performed significantly worse than controls in other psychometric tests. We therefore analyzed whether each subject showed impaired performance in the parameters analyzed by the other psychometric tests.

To identify the NAFLD patients showing impaired performance in each parameter measured by these tests, we applied the mean ± 2 SD criterion [21,27,28,29], calculating the mean and SD of the value for each parameter for the control group and adding or subtracting twice the SD to this mean to obtain a cutoff (see Table 2). This cutoff was applied to assess which parameters show impaired performance in each patient. Patients whose parameter score was within the range of the mean ± 2 SD were classified as without impairment and those falling outside this range were classified as having impaired performance for this parameter. This criterion has been previously used to identify patients with impaired performance in psychometric tests [21] and also to identify data outside the normal range in many other types of studies [27,28,29].

We used these data to design a new method to classify patients as with MCI or without MCI (NMCI) by developing a new score, as described in the results section.

### 2.5. Statistical Analysis

Values are given as mean ± SD, unless otherwise indicated. Results were analyzed by one-way ANOVA followed by post-hoc Tukey’s multiple comparison test. Variables were previously age-adjusted (except Stroop test) using univariate analysis of covariance (ANCOVA) with age included as a covariate, with the post-hoc Tukey test. For the other tests the parameters measured for each participant were adjusted for age according to test manual and Spanish normality tables. Chi-square test was applied to test differences in gender, dyslipemia, diabetes, arterial hypertension, and metabolic syndrome. Analyses were performed using GraphPad Prism version 8.0 (GraphPad Software, Inc., San Diego, CA, USA) and SPSS version 24.0 (SPSS Inc., Chicago, IL, USA), and two-sided *p* values < 0.05 were considered significant.

## 3. Results

### 3.1. Patients with NAFLD Show Neurological Impairment

Patients with NAFLD showed worse performance than controls in most parameters evaluated in the different tests (Table 2). The assessment of coordination shows that NAFLD patients need more time to perform the bimanual and visual-motor tasks than controls, although the difference is only significant for the bimanual task, indicating impaired bimanual coordination (Table 2). Non-alcoholic fatty liver disease also affects several cognitive functions.

In the d2 Test, NAFLD patients showed lower total number of characters processed (TR), total right answers (RA), and reduced mental concentration (CON) and effectiveness in the task (TOT) compared to controls. NAFLD patients show a significant increase in total errors (O + C) compared to controls (Table 2). The results obtained in the d2 test indicate that NAFLD patients show impaired selective/sustained attention and mental concentration, speed and amount of work done, accuracy of this work, and relationship between speed and accuracy.

In the Stroop Test, NAFLD patients performed a lower number of items than controls in all three (Congruent, Neutral, and Incongruent) tasks (Table 2), indicating impaired selective attention, psychomotor speed, cognitive flexibility, and inhibitory mental control.

In the Oral SDMT test, NAFLD patients obtained fewer total items and correct pairings compared to controls (Table 2), indicating a deficit in selective attention, psychomotor speed, cognitive flexibility, and inhibitory control.

NAFLD patients obtained worse results than controls in the Digit Span test, in both forward and backward forms, as well as in the total score (Table 2), which indicates declined selective verbal attention and working memory, respectively.

In the number-letter test, NAFLD patients obtained a lower number of right answers than controls (Table 2), indicating impairment in working memory in NAFLD patients.

These data indicate that patients with NAFLD would have MCI, i.e., an impairment in cognitive function when compared to that expected based on each individual’s age and education, but not evident.

### 3.2. Performance in Psychometric Tests Is Not Significantly Different in Patients with NAFL or NASH

**Figure 1:**
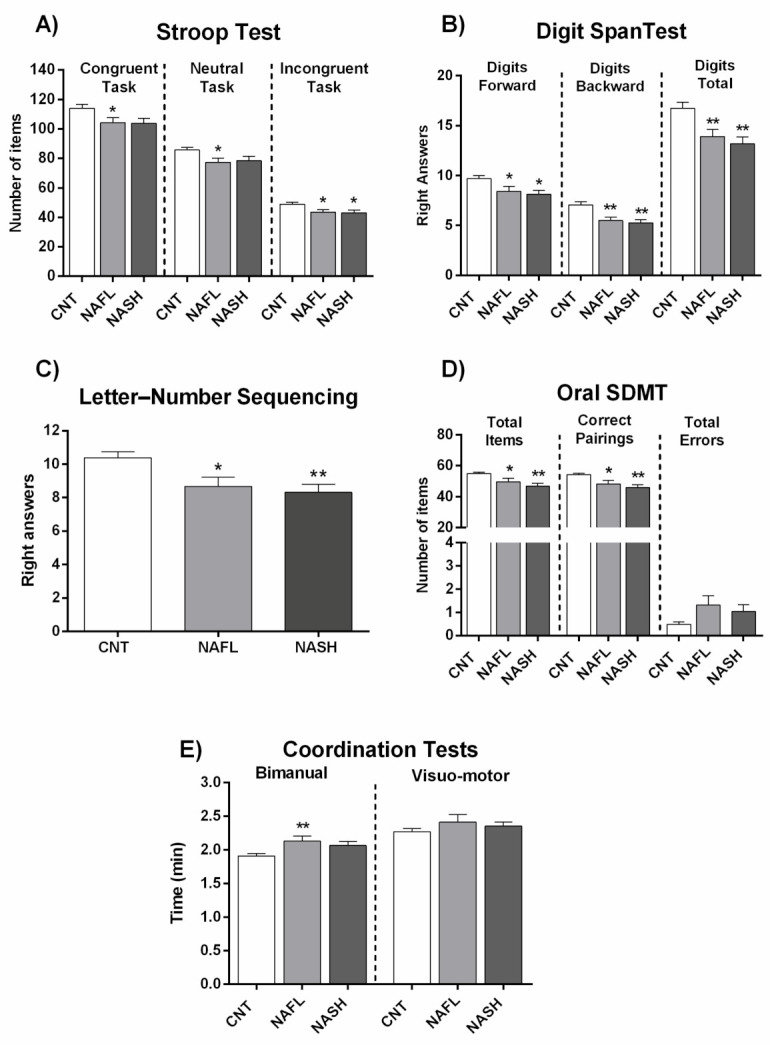
Performance of NAFL and NASH patients in psychometric tests. (**A**) Results of Congruent (number of words), Neutral (number of colors), and Incongruent (number of items completed) tasks in the Stroop test. (**B**) Results of the two tasks of Digit Span test (Digits forward and digits backward), and total Digit score. (**C**) Right answers in Letter-Number Sequencing test. (**D**) Performance in Oral SDMT (Symbol Digit Modalities Test) test. (**E**) Time (min) in Bimanual and Visuo-motor coordination tests. Results are the mean ± SEM (standard error of the mean). * *p* < 0.05; ** *p* < 0.01 vs. control group (CNT). NAFL, NASH, patients with nonalcoholic fatty liver and nonalcoholic steatohepatitis, respectively.

**Figure 2:**
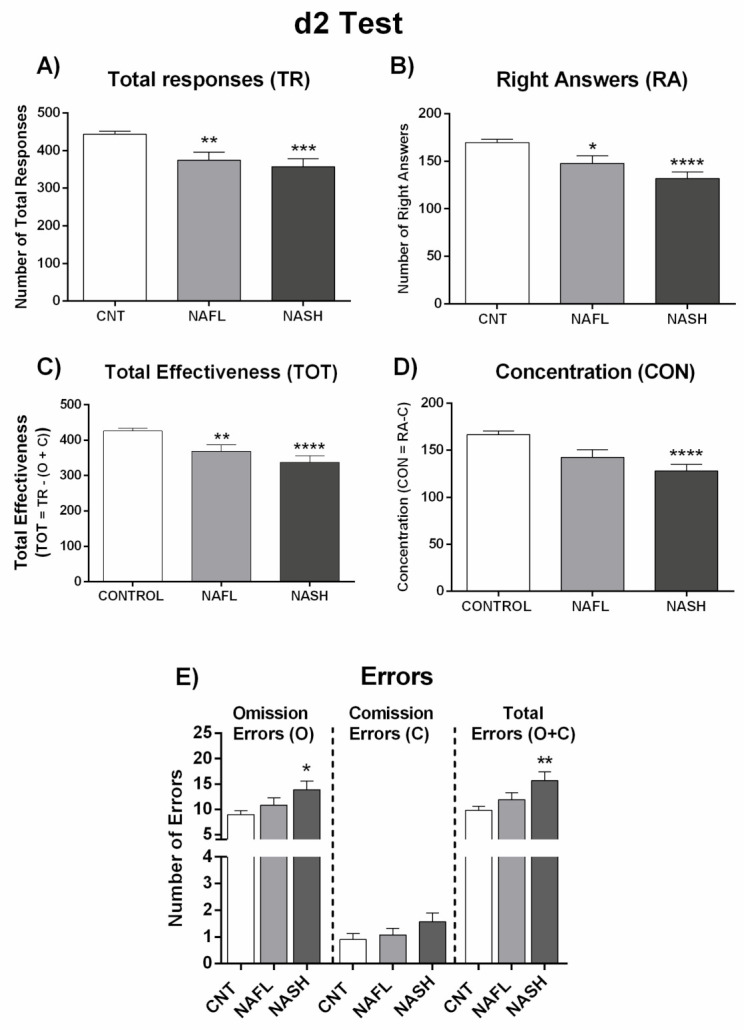
Performance of NAFL and NASH patients in d2 test. (**A**) Number of total responses (TR), (**B**) right answers (RA), and (**C**) total effectiveness (TOT), which was calculated by: TOT= TR − (O + C). (**D**) Concentration index (CON), which was calculated by: CON = RA − C. (**E**) Number of errors by Omission (O), Comission (C), and total errors (O + C). Results are the mean ± SEM. * *p* < 0.05; ** *p* < 0.01; *** *p* < 0.001; **** *p* < 0.0001 vs. control group (CNT). NAFL, NASH, patients with nonalcoholic fatty liver and nonalcoholic steatohepatitis, respectively.

To assess whether MCI appearance depends on progression of liver disease from NAFL to NASH, we analyzed performance in psychometric tests separately in these groups. Performance did not vary significantly between patients with NAFL and NASH in any of the psychometric test parameters (Figure 1 and Figure 2, Table S1), indicating that the progression of liver disease is not the main contributor to appearance of MCI. As there are no notable differences between NAFL and NASH patients in performance in psychometric tests, we categorized all NAFLD patients as a single group to classify them as with or without MCI.

### 3.3. Classification of NAFLD Patients as MCI or NMCI

The values reported in Table 2 and Table S1 are mean values for all patients and indicate that, as a whole, NAFLD patients performed worse than controls in most psychometric tests. However, we observed individual variability within this patient subset, some of them performing nearly as well as controls while others showing impaired performance.

A main aim of the study was to develop a score to detect and classify MCI/NMCI in NAFLD patients. As a reference, we first used the PHES, a battery of five psychometric tests used to detect mild cognitive impairment (MHE) in cirrhotic patients. According to the PHES, only 7 out of the 59 NAFLD patients (12%) met the criterion for MCI. However, a larger percentage of patients show impaired performance in many parameters of the other tests reported in Table 2.

To calculate the percentage of NAFLD patients with impaired performance in each parameter we used the mean ± 2 SD criterion (see Methods). Using the cutoff obtained for each parameter (Table 2), we calculated the proportion of NAFLD patients showing impaired performance in each task or parameter.

The test with the highest proportion of patients showing impairment was the Oral SDMT test with 31% and 32% of patients below the cutoff in total responses and correct pairing parameters, respectively. The other test failed by a large proportion of patients was the d2 test, which was failed by 29% of NAFLD patients in right answers, total responses, and mental concentration. NAFLD patients showed mild impairment in the Stroop test: 12% failed in both the congruent and the neutral tasks, and 5% in the incongruent task. Likewise, impairment rates were low in coordination tasks (14% in bimanual and 11% in visual-motor tests) and in the number-letter test (7%). Finally, no significant deficit was found among NAFLD patients in the Digit Span test, with only 2% of patients with impairment (Table 2).

### 3.4. Designing a New Score to Unveil Mild Cognitive Impairment in NAFLD Patients

The above results show that NAFLD patients have alterations in cognitive and motor function, which are not evident but can be unveiled using psychometric tests evaluating specific neurological functions. It would therefore be useful to select a few psychometric tests thatcould help identify which NAFLD patients are affected by MCI and might require appropriate monitoring. We next sought to pinpoint a combination of parameters arising from psychometric tests that would collectively provide a score to classify NAFLD patients as with (MCI) or without (NMCI) mild cognitive impairment. To develop the score, we selected three parameters according to two criteria: They must be affected in a large percentage of NAFLD patients and must assess different aspects of neurological function. The combination of parameters selected to develop this score were the RA and CON parameters of the d2 Test, failed by 29% of patients, and correct pairing of the Oral SDMT, failed by32% of patients (in bold in Table 2).

According to this score, MCI would be defined as an impaired performance in two or more of these three parameters. The scores showed that 19 of the 59 NAFLD patients in the study had poor performance in at least two parameters. This new score was therefore able to diagnose 32% of patients with MCI while the remaining 68% (40 of 59) were classified as NMCI.

Applying this new score separately to NAFL and NASH patients, 12 out of 33 (36%) NAFL patients, and 7 out of 26 (27%) NASH patients were classified as MCI. There were no significant differences in the prevalence of MCI in NAFL and NASH patients (*p* = 0.576; Table 1), thus confirming a similar prevalence in the two groups not directly linked to grade of liver disease.

### 3.5. Influence of Diabetes, Hypertension, Dyslipemia, and Metabolic Syndrome on the Prevalence of MCI in Patients with NAFLD

As shown in Table 1, NAFLD patients show higher Body Mass Index and a higher proportion of dyslipemia, diabetes, arterial hypertension, and metabolic syndrome than control subjects. However, no significant differences were found in these parameters between NAFLD patients with or without MCI (Table 1).

The data summarized in Table 3 show that the presence of diabetes and metabolic syndrome enhances the prevalence of MCI to 38 and 37% of NAFLD patients compared to 27 and 28% in the absence of these comorbidities. Hypertension or dyslipemia did not seem to significantly affect the prevalence of MCI in these patients (Table 3).

### 3.6. Performance in the Different Psychometric Tests of NAFLD Patients Classified as with or without MCI by the New Score

**Figure 3:**
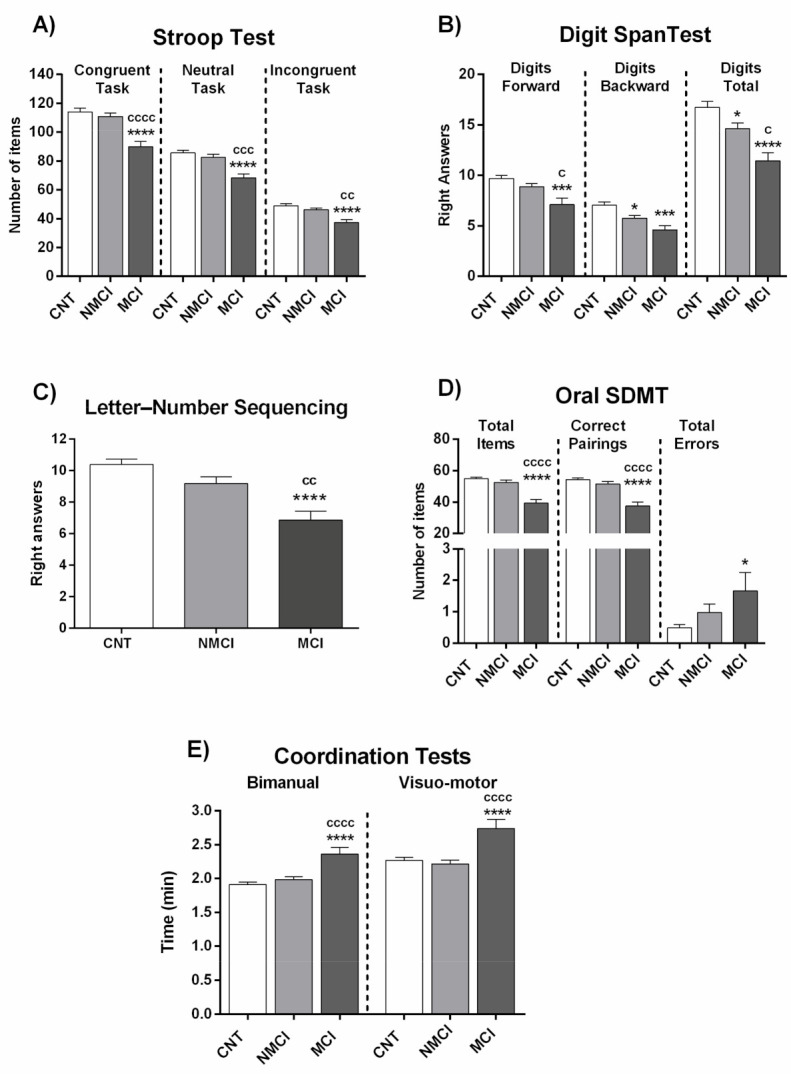
Performance in different psychometric tests of NAFLD patients classified by the new score as with (MCI) or without (NMCI) mild cognitive impairment. (**A**) Results of Congruent (number of words), Neutral (number of colors), and Incongruent (number of items completed) tasks in Stroop test. (**B**) Results of the two tasks of Digit Span test (Digits forward and digits backward), and total Digits score. (**C**) Right answers in Letter-Number Sequencing test. (**D**) Performance in Oral SDMT test. (**E**) Time (min) in Bimanual and Visuo-motor coordination tests. Results are the mean ± SEM. * *p* < 0.05; *** *p* < 0.001; **** *p* < 0.0001 vs. control group (CNT). ^c^ *p* < 0.05; ^cc^ *p* < 0.01; ^ccc^ *p* < 0.001; ^cccc^ *p* < 0.0001, NASH MCI vs. NASH NMCI.

**Figure 4:**
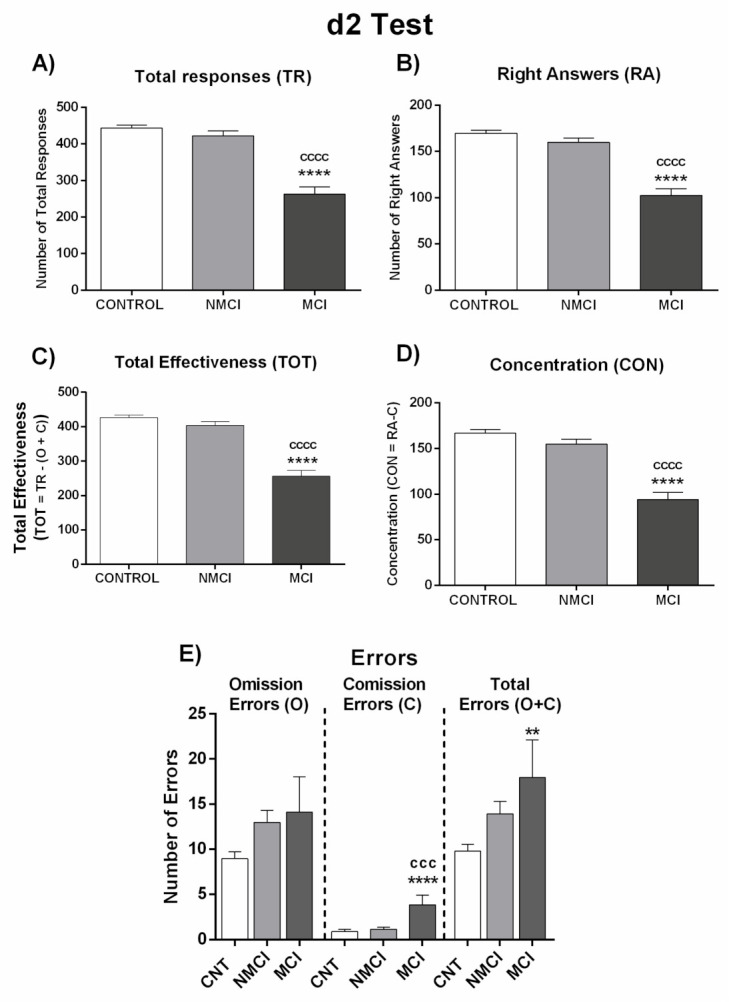
Performance of NAFLD patients classified by the new score as with (MCI) or without (NMCI) mild cognitive impairment in d2 test. (**A**) Number of total responses (TR), (**B**) right answers (RA), and (**C**) total effectiveness (TOT), which was calculated by: TOT= TR − (O + C). (**D**) Concentration index (CON), which was calculated by: CON = RA − C. (**E**) Number of errors by Omission (O), Comission (C), and total errors (O + C). Results are the mean ± SEM. ** *p* < 0.01; **** *p* < 0.0001 vs. control group (CNT). ^ccc^ *p* < 0.001; ^cccc^ *p* < 0.0001, NASH MCI vs. NASH NMCI.

Once the patients were classified as MCI or NMCI according to this new score, we analyzed the alterations in different neurological functions in each patient group compared to healthy subjects (Figure 3 and Figure 4; Table S2).

In the Stroop test, NAFLD patients with MCI correctly named fewer words, colors, and items than NMCI NAFLD patients and controls (Figure 3A; Table S2). This indicates that NAFLD patients with MCI show impaired selective attention, psychomotor velocity, cognitive flexibility, and inhibitory mental control as measured by the Stroop test. NMCI patients showed no differences performing these tasks compared to controls (Figure 3A; Table S2).

Concerning the Digit Span test, in the forward version of the test NAFLD patients with MCI repeated a lower number of series than controls or NMCI NAFLD patients, reaching statistical significance when compared to NMCI patients and controls (Figure 3B; Table S2). Therefore, MCI NAFLD patients show impaired selective attention (verbal attention) measured by this test. In the backward version, MCI NAFLD patients remembered a lower number of series than controls and NMCI NAFLD patients. The Digits total score was lower in MCI patients than in NMCI patients and controls. Patients with NMCI also showed statistical differences (*p* < 0.05) compared to the control group. This points to impaired working memory in MCI NAFLD patients (Figure 3B; Table S2). A similar impairment in working memory was also observed with the Number-letter Test, in which MCI NAFLD patients gave fewer right answers than controls or NMCI NAFLD patients (Figure 3C; Table S2).

The Oral SDMT test reveals strong alterations in NAFLD patients with MCI (but not in NMCI patients) in both total items and correct pairings parameters compared to controls and NMCI NAFLD patients (Figure 3D; Table S2).

Coordination was also impaired in NAFLD patients with MCI but not in those with NMCI. MCI patients needed more time to finish the tasks than controls or NMCI patients, in both the bimanual test and visual-motor test (Figure 3E; Table S2).

Performance in the d2 test was also impaired in patients with MCI but not in those with NMCI. Total responses (TR), right answers (RA), TOT (total correctly processed), and CON (concentration performance) were significantly reduced in MCI patients compared to NMCI patients and controls. These results show lower selective/sustained attention and mental concentration, speed or amount of work done, and accuracy of the work in patients with NAFLD and MCI but not in those with NMCI (Figure 4; Table S2).

**Figure 5:**
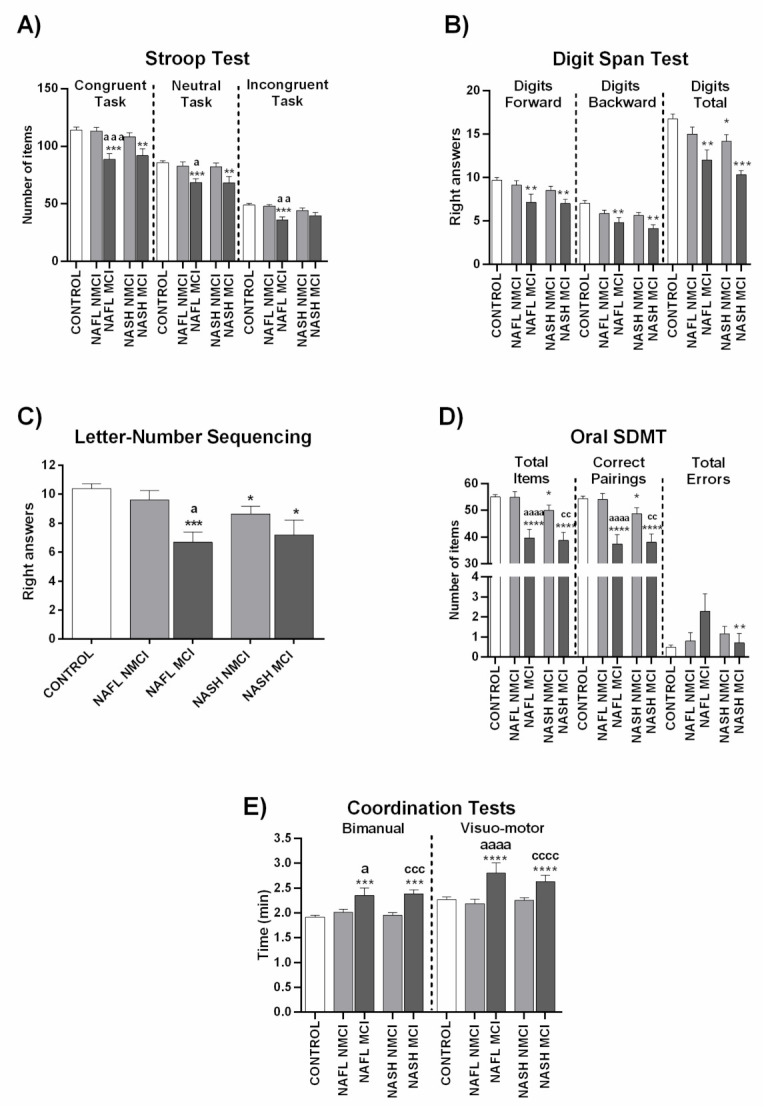
Performance in different psychometric tests of NAFL and NASH patients classified by the new score as with (MCI) or without (NMCI) mild cognitive impairment. (**A**) Results of Congruent (number of words), Neutral (number of colors), and Incongruent (number of items completed) tasks in Stroop test. (**B**) Results of the two tasks of Digit Span test (Digits forward and digits backward), and total Digits score. (**C**) Right answers in Letter-Number Sequencing test. (**D**) Performance in Oral SDMT test. (**E**) Time (min) in Bimanual and Visuo-motor coordination tests. Results are the mean ± SEM. * *p* < 0.05; ** *p* < 0.01; *** *p* < 0.001; **** *p* < 0.0001 vs. control group (CNT), ^a^ *p* < 0.05; ^aa^ *p* < 0.01; ^aaa^ *p* < 0.001; ^aaaa^ *p* < 0.0001, NAFL MCI vs. NAFL NMCI; ^cc^ *p* < 0.01; ^ccc^ *p* < 0.001; ^cccc^ *p* < 0.0001, NASH MCI vs. NASH NMCI.

**Figure 6:**
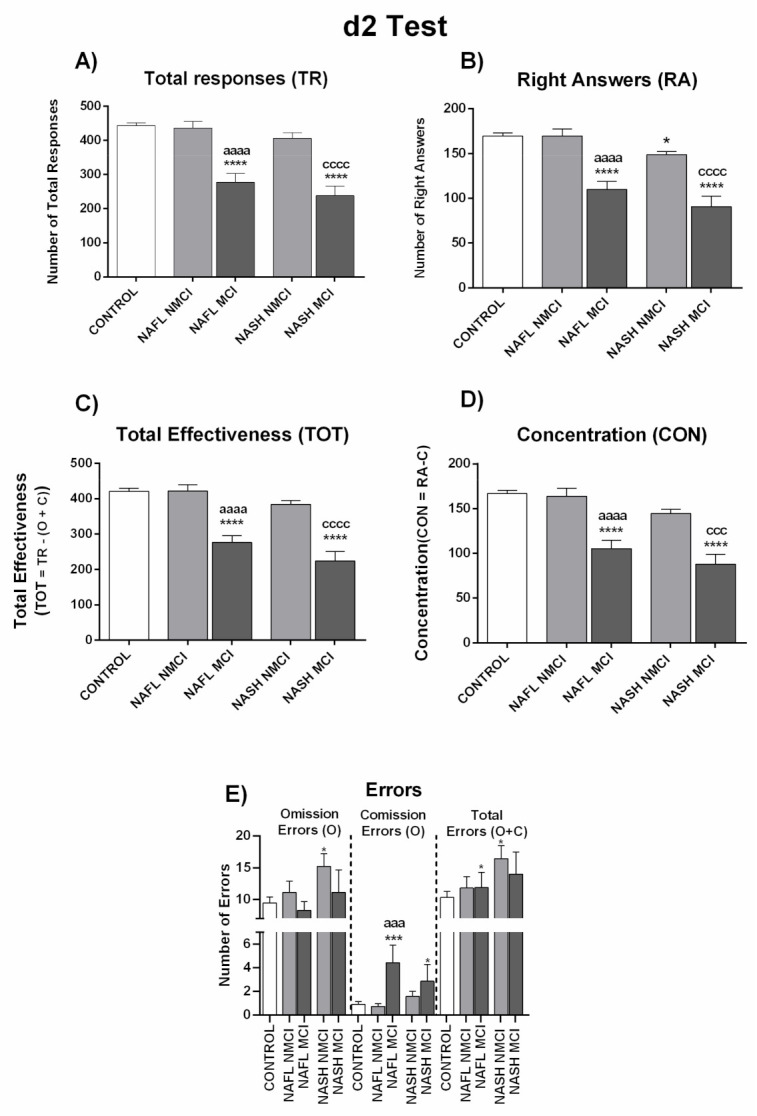
Performance of NAFL and NASH patients classified by the new score as with (MCI) or without (NMCI) mild cognitive impairment in d2 test. (**A**) Number of total responses (TR), (**B**) right answers (RA), and (**C**) total effectiveness (TOT), which was calculated by: TOT= TR − (O + C) (**D**) Concentration index (CON), which was calculated by: CON = RA − C. (**E**) Number of errors by Omission (O), Comission (C), and total errors (O + C). Results are the mean ± SEM. * *p* < 0.05; *** *p* < 0.001; **** *p* < 0.0001 vs. control group (CNT), ^aaa^ *p* < 0.001; ^aaaa^ *p* < 0.0001, NAFL MCI vs. NAFL NMCI; ^ccc^ *p* < 0.001; ^cccc^ *p* < 0.0001, NASH MCI vs. NASH NMCI.

Figure 5 and Figure 6 and Table S3 show the performance of NAFL and NASH patients with or without MCI in each test. Again, the data indicate that MCI appearance is not directly dependent on grade of liver damage.

## 4. Discussion

This report shows that patients with NAFLD may present with MCI before reaching liver cirrhosis. NAFLD patients show impaired selective and sustained attention, mental concentration, speed, amount, and accuracy of work done as assessed in the d2 test; impairment in selective attention, psychomotor speed, cognitive flexibility, and inhibitory mental control in the Stroop Test; a deficit in selective attention, psychomotor speed, cognitive flexibility, and inhibitory control in the Oral SDMT test; impairment in selective verbal attention and working memory in the Digit Span test; and in working memory in the Number-Letter test. Bimanual and visual-motor coordination are only mildly affected.

Prevalence of MCI is higher than suspected in these patients. To bring to light this MCI, we have developed a new score thatdetects MCI with more sensitivity than the PHES used to identify MHE in cirrhotic patients. Using the PHES, 12% of NAFLD patients (7 out of 59) were classified as having MCI. This prevalence is lower than in cirrhotic patients, who show MHE prevalence of 31–59% when diagnosed using the PHES [13,21,30,31]. However, it has been reported that the PHES is not sensitive enough and fails to detect the presence of mild cognitive impairment in a substantial proportion of cirrhotic patients. This MCI can be detected using other psychometric tests evaluating different aspects of neurological function [9,20,21]. We have previously shown that Oral Symbol Modalities Test (SDMT), d2 and bimanual and visual-motor coordination tests were more sensitive than PHES in detecting these early alterations in cirrhotic patients [21], prompting us to use these particular tests to evaluate MCI in patients with NAFLD. Impaired performance had a prevalence ranging between 0 and 32% in each individual test, as analyzed using the mean ± 2 SD criterion. The neurological functions most frequently affected in NAFLD patients were the speed of mental processing in selective attention tasks, evaluated by correct pairings in the Oral SDMT test (impaired in 32% of patients), precision of mental processing in selective/sustained attention tasks, evaluated in the d2 test by right answers (impaired in 29% of patients), and concentration, evaluated by the d2 test (impaired in 29% of patients).

To improve the identification of NAFLD patients showing MCI, we developed a new score taking into account the performance of the patients in the following three parameters: RA (right answers) and CON (concentration) of the d2 test, and correct pairings of the Oral SDMT test. Patients who show impaired performance in two or more of these tests are considered to have MCI. This new score classified 19 of the 59 NAFLD patients (32% of patients) as with MCI, confirming its far greater sensitivity than the PHES in detecting MCI in NAFLD patients.

The prevalence of MCI in NAFLD patients is very high, highlighting the benefit of performing psychometric tests in these patients to uncover this MCI and take appropriate measures to reverse and prevent the progression of MCI in NAFLD patients.

It is worth noting that the appearance of MCI in NAFLD is not directly dependent on the grade of liver disease. As described in the Methods section, patients were classified as NAFL or NASH according to liver disease grade. We evaluated whether the presence or grade of MCI was different in patients with NAFL or NASH, observing no significant performance differences in the different psychometric tests between the two patient groups (Figure 1 and Figure 2; Table S1). For this reason, all NAFLD data are presented here together, classified only as MCI or NMCI.

This finding indicates that the appearance of MCI in NAFLD depends on other factors additional to grade of liver disease. This concurs with results reported in cirrhotic patients by Mangas-Losada et al. [32] who found that rather than the grade of liver disease (MELD or Child-Pugh), MHE appearance depends on a shift in the type of peripheral inflammation.

We assessed the possible influence of some comorbidities on the prevalence of MCI in NAFLD patients. The data obtained show that the presence of diabetes enhances the prevalence from 27% in the absence of diabetes to 38% in its presence while the presence of metabolic syndrome enhances MCI prevalence from 28% in its absence to 37% in its presence. Hypertension or dyslipemia did not seem to significantly affect the prevalence of MCI in these patients (Table 3).

Although the prevalence of MCI is similar between patients with NAFL and NASH or between cirrhotic patients with different MELD or Child-Pugh scores, the progression of liver disease from NAFLD to cirrhosis is associated with a notable increase in the prevalence of MCI (or MHE). As mentioned above, the prevalence of MCI detected by the PHES is 12% in NAFLD but is significantly higher (30–59%) in cirrhotic patients. Concerning the overall prevalence of mild cognitive impairment, the new score developed classifies 32% of NAFLD patients as having MCI, a prevalence similar for patients with NAFL (36%) or NASH (27%). The application of a similar score would classify around 65% of cirrhotic patients with MCI [21]. These data indicate that small differences in the grade of liver disease within NAFLD patients with NAFL and NASH or within cirrhotic patients with Child-Pugh A or B do not affect MCI appearance or grade; however, progression from NAFLD to cirrhosis clearly increases the risk for MCI, likely due to changes in processes involved in triggering MCI-MHE such as inflammation, immunophenotype, or neuroinflammation [32,33,34]. It should be also noted that the appearance of MHE in cirrhotic patients or mild cognitive impairment in NAFL and NASH patients does not depend mostly on the grade but on the type of inflammation. For example, cirrhotic patients in general show strong inflammation; however, 35% of them do not show MHE, in spite of high levels of inflammatory markers. The appearance of MHE is triggered by a shift in the type of inflammation, to an “autoimmune-like” form, with increased activation and differentiation of CD4 lymphocytes to Th22,Thf, and Th17 [32]. Concerning NAFL and NASH patients, although patients with NASH could have more inflammation than NAFL patients (but less than cirrhotic patients) this is not enough to induce MCI unless a shift in the type of inflammation also occurs in a similar way to that present in cirrhotic patients with MHE.

It is also noteworthy that NAFLD and cirrhotic patients seem to have different patterns of affected neurological functions. The two most affected functions in cirrhotic patients are bimanual coordination with impairment in 51% and visual-motor coordination impaired in 43% of patients [21]. However, we found that these functions are deficient in only 14% or 11% of NAFLD patients, which indicates that the prevalence of incoordination is very low in these patients, but that this impairment becomes much more common with progression to cirrhosis.

The prevalence of mild cognitive impairment is also higher in patients with liver cirrhosis than in NAFLD patients, but the differences are smaller. For example, concentration as evaluated by the d2 test is diminished in 49% of cirrhotic patients and in 29% of NAFLD patients. The total correct values in the d2 test are impaired in 51% of cirrhotic patients and in 29% of NAFLD patients and the total items in the Oral SDMT test in 54% of cirrhotic patients and in 29% of NAFLD patients.

This suggests that mild cognitive impairment would appear at early stages of chronic liver disease, while motor incoordination would develop later on.

We have previously shown that the earliest neuropsychological alterations are not the same for all cirrhotic patients. In a subgroup of patients, alterations in coordination appear before mild cognitive impairment while in another sub-group, concentration and attention deficits emerge earlier than coordination impairment. This is clearly illustrated by the classification of patients into different clusters or subgroups according to types of tests failed [21]. This heterogeneity in the first neuropsychological alterations to manifest in cirrhotic patients is also supported by data from Butz et al. [20], Felipo et al. [35], and Montagnese et al. [36].

A similar heterogeneity is observed in the earliest alterations in patients with NAFLD, as highlighted by the fact that 7 out of the 59 patients are classified as MCI by the PHES and 19 by the new score. However, three of the seven patients categorized as MCI by the PHES are classified as NMCI by the new score, indicating that they have worse performance in neurological functions, which are not evaluated by the three parameters included in the new score. This also shows that prevalence of MCI in NAFLD is slightly higher than the 32% detected by the new score, reaching 37% if we add the patients detected by the PHES. Although performing a wider battery of psychometric tests would detect MCI in NAFLD patients with higher sensitivity, this would be time consuming and possibly unrealistic in clinical practice. This new score is more practical for clinical practice, as tests can be performed in only 7 min and still detect MCI with high sensitivity.

The above data also support the idea that the earliest neurological changes vary among different individual patients, therefore necessitating a combination of tests to detect NAFLD patients with MCI with enough sensitivity. We provide herein a combination of three parameters, measuring different neuropsychological alterations, which accurately detect MCI in NAFLD patients.

It is estimated that over 64 million people have NAFLD in the United States. Taking into account only four countries (Germany, France, Italy, and United Kingdom), there are around 52 million people with NAFLD in Europe [37]. The 32% prevalence of MCI, as detected by the new proposed score, implies that around 21 million people in USA and 17 million in these four European countries have NAFLD-related MCI. The features of this MCI are very similar to those of MHE present in cirrhotic patients, affecting similar neurological functions, and would consequently have a similar effect on ability to perform daily life tasks requiring attention and concentration. This would reduce quality of life and also predispose patients to progression to worse stages of cognitive and motor impairment. As is the case for MHE in cirrhotic patients, early detection of MCI in NAFLD patients enables measures to be taken that could reverse and/or prevent progression of neurological dysfunction, as well as associated complications and adverse outcomes. The new score described in this work will be very useful in clinical practice to identify MCI in patients with NAFLD who attend the outpatient consultation of Gastroenterology/Hepatology Services or in other clinical settings.

## 5. Conclusions

Patients with NAFLD may present with mild cognitive impairment (MCI) before reaching liver cirrhosis, showing impairment of selective, sustained, and verbal attention, mental concentration, psychomotor speed, cognitive flexibility, inhibitory mental control, and working memory. We have developed a new, rapid (7 min), and sensitive score to detect MCI in NAFLD patients. This work shows that MCI prevalence is significant in NAFLD patients. Psychometric testing is warranted in these patients to unveil MCI and take appropriate measures to reverse and prevent its progression.

## Figures and Tables

**Table 1:** Patients characteristics.

PARAMETER	CONTROLS (x ± SD)	NAFLD Patients without MCI (x ± SD)	NAFLD Patients with MCI (x ± SD)	Global *p*-Values
Number of subjects (N)	53	40	19	
Gender (Male/Female)	29M/24F	22M/18F	8M/11F	0.598
Age (years)	59 ± 8	58 ± 7	59 ± 11	0.935
Education (years)	14 ± 4	14 ± 4	12 ± 5	0.424
Biopsy (N)	-	31	13	
Fibroscan (N)	-	8	5	
NAS (0–3/4–8)	-	11/16	5/6	
FAST (cut off ≥0.35)	-	7/1	5/0	
Fibrosis grade (F0/F1/F2/F3)	-	6/5/16/4	2/5/5/1	0.450
NAFL (N)	-	21	12	0.576
NASH (N)	-	19	7	
LSM (kPa)	-	8.7 ± 4.4	9.2 ± 4.8	0.777
CAP (dB/M)	-	332 ± 41	310 ± 59	0.170
AST (U/L)	24 ± 5	38 ± 18 ***	33 ± 11	0.0003
ALT (U/L)	25 ± 8	50 ± 24 ***	42 ± 20 *	<0.0001
Bilirrubin (mg/dL)	0.6 ± 0.2	0.7 ± 0.4	0.6 ± 0.5	0.427
Platelets (×10^9^)	229 ± 41	228 ± 75	255 ± 66	0.369
INR	1.02 ± 0.03	1.03 ± 0.06	1.02 ± 0.06	0.798
BMI (Body Mass Index)	27.7 ± 4	31 ± 4 *	32 ± 7	0.04
Dyslipemia (YES/NO)	13 YES/40 NO	26 YES/14 NO	11 YES/8 NO	0.0002
Diabetes (YES/NO)	8 YES/45 NO	18 YES/22 NO	11 YES/8 NO	0.0004
Arterial Hypertension (YES/NO)	11 YES/42 NO	22 YES/18 NO	11 YES/8 NO	0.0007
Metabolic Syndrome (YES/NO)	7 YES/46 NO	19 YES/21 NO	11 YES/8 NO	<0.0001

ALT: Alanine aminotransferase; AST: Aspartate aminotransferase; CAP: Controlled attenuation parameter; FAST: FibroScan-AST; INR: International normalized ratio; LSM: Liver stiffness measurement; MCI: Mild cognitive impairment; NAFLD: Nonalcoholic fatty liver disease; NAFL: Non-alcoholic fatty liver; NASH: Non-alcoholic steatohepatitis; NAS: NASH Activity Score; SD: Standard deviation; x: Mean. Data were analyzed by one-way ANOVA followed by post-hoc Tukey’s multiple comparison test: * *p* < 0.05; *** *p* < 0.001; vs. control group. Chi-square test was applied to test differences in gender, dyslipemia, diabetes, arterial hypertension, and metabolic syndrome. No significant differences were found between NAFLD patients without or with MCI.

**Table 2:** Cutoff and proportion of NAFLD patients showing cognitive impairment for each psychometric test parameter.

TEST (Parameter)	CONTROLS (x ± SD)	2 SD	Cut off Impairment (x ± 2 SD)	NAFLD Patients (x ± SD)	% of Patients with Impairment
Bimanual coordination (min)	1.91 ± 0.26	0.52	2.43	2.11 ± 0.37 **	14
Visual-motor coordination (min)	2.27 ± 0.32	0.64	2.91	2.39 ± 0.50	11
**d2 Test**					
TR Values	444 ± 55	110	334	367 ± 110 ****	29
RA Values	170 ± 24	48	121	141 ± 40 ****	**29**
O Values	9 ± 5	13	23	13 ± 12 *	20
C Values	0.9 ± 1.6	3.23	4.1	2.1 ± 3.2 *	15
O + C Values	10 ± 5	13	24	15 ± 13 **	24
TOT Values	426 ± 52	128	548	355 ± 97 ****	25
CON Values	167 ± 27	54	221	134 ± 45 ****	**29**
**Stroop Test**					
Congruent Task (Number of words)	114 ± 18	36	78	104 ± 18 **	12
Neutral Task (Number of colors)	86 ± 13	26	60	78 ± 15 **	12
Incongruent Task (Number of items)	49 ± 10	20	28	43 ± 9 **	5
**Oral SDMT test**					
Total items	55 ± 6	13	42	48 ± 11 ***	31
Correct pairings	54 ± 7	13	41	47 ± 12 ***	**32**
Errors	0.5 ± 0.7	1.37	1.85	1.3 ± 2.1 *	15
**DIGIT SPAN Test**					
Digits forward (right answers)	10 ± 2	4	5	8 ± 2 **	2
Digits backward (right answers)	7 ± 2	5	2	5 ± 2 ****	0
Digits Total Score	17 ± 4	8	9	14 ± 4 ****	2
NUMBER-LETTER Test (right answers)	10 ± 2	5	5	9 ± 3 ***	5

x: Mean; SD: Standard deviation; NAFLD, nonalcoholic fatty liver disease; Oral SDMT, Symbol digit modalities test (oral version). TR, Total number of characters processed; TOT, Total correctly processed; CON, Concentration performance; RA, Total right answers; O, errors of omission; C, errors of commission; O + C, Total errors. Differences between NAFLD patients compared with the controls are indicated by *: * *p* < 0.05; ** *p* < 0.01; *** *p* < 0.001; **** *p* < 0.0001. Parameters selected to develop the new score are in bold.

**Table 3:** Influence of diabetes, hypertension, dyslipemia, and metabolic syndrome on the prevalence of MCI in patients with NAFLD.

Patients	Patients with MCI	Total Patients	Prevalence of MCI (%)
Total	19	59	32
With diabetes	11	29	38
Without diabetes	8	30	27
With hypertension	11	33	33
Without hypertension	8	26	31
With dyslipemia	11	37	30
Without dyslipemia	8	22	36
With metabolic syndrome	11	30	37
Without metabolic syndrome	8	29	28

MCI: Mild cognitive impairment; NAFLD: Nonalcoholic fatty liver disease.

## Data Availability

All data generated or analyzed during this study are included in this published article and Supplementary material.

## Supplementary Materials

The following are available online: at https://www.mdpi.com/article/10.3390/jcm10132806/s1, Table S1: Performance of NAFL and NASH patients in psychometric tests, Table S2: Performance in different psychometric tests of NAFLD patients classified by the new score as with or without mild cognitive impairment, Table S3: Performance of NAFL and NASH patients with and without MCI in different psychometric tests.

## Abbreviations

NAFLD	nonalcoholic fatty liver disease
MCI	mild cognitive impairment
NAFL	nonalcoholic fatty liver
NASH	nonalcoholic steatohepatitis
PHES	psychometric hepatic encephalopathy score
MHE	minimal hepatic encephalopathy
NMCI	without MCI
