# Supplementary material for: A New Score Unveils a High Prevalence of Mild Cognitive Impairment in Patients with Nonalcoholic Fatty Liver Disease
Source: J Clin Med. 2021 Jun 25;10(13):2806. doi: 10.3390/jcm10132806 (PMC8268962; doi:10.3390/jcm10132806)
Supplement: Supplementary file 1 [file jcm-10-02806-s001.zip › jcm-1237182-supplementary.pdf]

A New Score Unveils a High Prevalence of Mild Cognitive Impairment in Patients with Nonalcoholic Fatty Liver Disease

Carla Giménez-Garzó, Alessandra Fiorillo, María-Pilar Ballester-Ferré, Juan-José Gallego, Franc Casanova-Ferrer, Amparo Urios, Salvador Benlloch, David Martí-Aguado, Teresa San-Miguel, Joan Tosca, María-Pilar Ríos, Cristina Montón, Lucía Durbán, Desamparados Escudero-García, Luis Aparicio, Vicente Felipo, Carmina Montoliu

Supplementary Tables

Table S1. Performance of NAFL and NASH patients in psychometric tests.

TEST (parameter)	CONTROLS (x ± SD)	NAFL patients (x ± SD)	NASH patients (x ± SD)
Subject Number (N)	53	33	26
Age	59 ± 8	57 ± 10	59 ± 7
Bimanual coordination (min)	1.91 ± 0.26	2.13 ± 0.41	2.07 ± 0.30
Visual-motor coordination (min)	2.32 ± 0.39	2.42 ± 0.61	2.35 ± 0.31
**d2 Test**			
TR Values	444 ± 55	375 ± 116**	357 ± 104***
RA Values	170 ± 24	148 ± 43*	132 ± 34***
O Values	9 ± 5	11 ± 7	14 ± 8*
C Values	0.9 ± 1.6	1.1 ± 31.3	1.6 ± 1.6
O+C Values	10 ± 5	12 ± 7	16 ± 8**
TOT Values	426 ± 52	369 ± 101**	337 ± 92****
CON Values	167 ± 27	142 ± 46*	128 ± 34****
**Stroop Test**			
Congruent Task (Number of words)	114 ± 18	104 ± 20*	104 ± 17
Neutral Task (Number of colours)	86 ± 13	77 ± 16*	78 ± 15
Incongruent Task (Number of items)	49 ± 10	44 ± 10*	43 ± 9*
**Oral SDMT test**			
Total items	55 ± 6	50 ± 12*	47 ± 10**
Correct pairings	54 ± 7	48 ± 13*	46 ± 10**
Errors	0.5 ± 0.7	1.3 ± 2.3	1.0 ± 1.5
**DIGIT SPAN Test**			
Digits forward (right answers)	10 ± 2	8 ± 3*	8 ± 2*
Digits backward (right answers)	7 ± 3	5 ± 2**	5 ± 1**
Digits Total Score	17 ± 4	14 ± 4**	13 ± 3**
Number-Letter Test (right answers)	10 ± 2	9 ± 3*	8 ± 2**

SD: standard deviation; x: mean; NAFL: non-alcoholic fatty liver; NASH: Non-alcoholic steatohepatitis; Oral SDMT, Symbol digit modalities test (oral version). TR, Total number of characters processed; TOT, Total correctly processed; CON, Concentration performance; RA, Total right answers; O, errors of omission; C, errors of commission; O+C, Total errors. Differences between NAFL and NASH patients compared with the controls are indicated by: *p<0.05; **p<0.01; ***p<0.001; ****p<0.0001.

Table S2. Performance in different psychometric tests of NAFLD patients classified by the new score as with or without mild cognitive impairment

TEST (parameter)	CONTROLS (x ± SD)	NAFLD patients without MCI (x ± SD)	NAFLD patients with MCI (x ± SD)
SubjectNumber (N)	53	40	19
Age	59 ± 8	58 ± 7	59 ± 11
Bimanual coordination (min)	1.91 ± 0.26	1.98 ± 0.25	2.36 ± 0.43****,cccc
Visuo-motor coordination (min)	2.32 ± 0.39	2.22 ± 0.33	2.74 ± 0.60****,cccc
PHES	2.32 ± 0.39	2.22 ± 0.33	2.74 ± 0.60****,cccc
**d2 Test**			
TR Values	444 ± 55	422 ± 78	263 ± 85****,cccc
TA Values	170 ± 24	160 ± 28	102 ± 31****,cccc
O Values	9 ± 5	13 ± 7	14 ± 17
C Values	0.9 ± 1.6	1.1 ± 1.4	3.8 ± 4.6****,ccc
O+C Values	10 ± 5	14 ± 8	18 ± 18**
TOT Values	426 ± 52	404 ± 67	256 ± 70****,cccc
CON Values	167 ± 27	155 ± 33	94 ± 36****,cccc
**Stroop Test**			
Congruent Task (Number of words)	114 ± 18	111 ± 15	90 ± 16****,cccc
Neutral Task (Number of colours)	86 ± 13	82 ± 15	68 ± 12****,ccc
Incongruent Task (Number of items)	49 ± 10	46 ± 9	37 ± 8****,cc
**Oral SDMT test**			
Total items	55 ± 6	53 ± 10	39 ± 10****,cccc
Correctpairings	54 ± 7	52 ± 10	38 ± 10****,cccc
Errors	0.5 ± 0.7	1.0 ± 1.7	1.7 ± 2.5*
**DIGIT SPAN Test**			
Digits forward (rightanswers)	10 ± 2	9 ± 2	7 ± 3***,c
Digitsbackward (rightanswers)	7 ± 3	6 ± 2*	5 ± 2***
Digits Total Score	17 ± 4	15 ± 3*	11 ± 3****,c
Number-letter Test (right answers)	10 ± 2	9 ± 3	7 ± 2****,cc

x: mean; SD: standard deviation; NAFLD: nonalcoholic fatty liver disease; MCI, mild cognitive impairment; Oral SDMT, Symbol digit modalities test (oral version). TR, Total number of characters processed; TOT, Total correctly processed; CON, Concentration performance; RA, Total right answers; O, errors of omission; C, errors of commission; O+C, Total errors. Differences between NAFLD patients compared with the controls are indicated by: *p<0.05; **p<0.01; ***p<0.001; ****p<0.0001. Differences between patients with and without MCI are indicated by: [c]p<0.05; [cc]p<0.01; [ccc]p<0.001; [cccc]p<0.0001.

Table S3. Performance of NAFL and NASH patients with and without MCI in different psychometric tests.

TEST (parameter)	CONTROLS (x ± SD)	NAFL patients NMCI (x ± SD)	NAFL patients MCI (x ± SD)	NASH patients NMCI (x ± SD)	NASH patients MCI (x ± SD)
Subject Number (N)	53	21	12	19	7
Age	59 ± 8	57 ± 7	58 ± 13	59 ± 7	60 ± 8
Bimanual coordination (min)	1.91 ± 0.26	2.01 ± 0.27	2.35 ± 0.53^{***,a}	1.95 ± 0.24	2.38 ± 0.21^{***,ccc}
Visuo-motor coordination (min)	2.27 ± 0.32	2.18 ± 0.41	2.80 ± 0.70$^{****, aaaa}$	2.25 ± 0.24	2.63 ± 0.35^{****,cccc}
PHES	0.42 ± 1.06	0.48 ± 1.57	-3.25 ± 3.70$^{****, aaaa}$	0.68 ± 1.45	-1.86 ± 1.57^{****,cccc}
**d2 Test**					
TR Values	443 ± 55	436 ± 84	277 ± 91$^{****, aaaa}$	406 ± 69	238 ± 75$^{****, cccc}$
RA Values	167 ± 24	170 ± 33	110 ± 29$^{****, aaaa}$	149 ± 16*	91 ± 31$^{****, cccc}$
O Values	9 ± 6	12 ± 7	16 ± 20	15 ± 7*	11 ± 9
C Values	0.9 ± 1.6	0.7 ± 0.9	4.4 ± 5.1$^{***, aaa}$	1.6 ± 1.7	2.9 ± 3.7*
O+C Values	10 ± 7	12 ± 7	20 ± 22*	16 ± 8*	14 ± 9
TOT Values	421 ± 64	422 ± 78	277 ± 63$^{****, aaaa}$	384 ± 47	224 ± 72$^{****, cccc}$
CON Values	167 ± 27	164 ± 40	106 ± 30$^{****, aaaa}$	145 ± 19	88 ± 29^{****,ccc}
**Stroop Test**					
Congruent (Number of words)	114 ± 18	113 ± 15	89 ± 17$^{***, aaa}$	108 ± 15	92 ± 16**
Neutral (Number of colours)	86 ± 13	83 ± 15	68 ± 12^{***,a}	82 ± 14	68 ± 14**
Incongruent (Number of items)	49 ± 10	48 ± 7	36 ± 9$^{***, aa}$	44 ± 10	40 ± 7
**Oral SDMT test**					
Total items	55 ± 6	55 ± 10	40 ± 11$^{****, aaaa}$	50 ± 9*	39 ± 8^{****,cc}
Correct pairings	54 ± 7	54 ± 10	37 ± 12$^{****, aaaa}$	49 ± 10*	38 ± 8^{****,cc}
Errors	0.5 ± 0.7	0.8 ± 1.8	2.3 ± 2.9	1.1 ± 1.6	0.7** ± 1.2
**DIGIT SPAN Test**					
Digits forward (right answers)	9.7 ± 2.1	9.1 ± 2.3	7.2 ± 3.2**	8.5 ± 2.0	7.0 ± 1.3**
Digits backward (right answers)	7.0 ± 2.4	5.8 ± 1.8	4.8 ± 2.0**	5.6 ± 1.4	4.1 ± 1.0**
Digits Total Score	16.7 ± 4.1	15.0 ± 3.07	12.0 ± 4.0**	14.2 ± 3.2*	10.3 ± 1.2***
Number-letter Test (right answers)	10.1 ± 2.4	9.6 ± 3.0	6.7 ± 2.2$^{***, a}$	8.6 ± 2.2*	7.2 ± 2.3*

x: mean; SD: standard deviation; NAFL: non-alcoholic fatty liver; NASH: Non-alcoholic steatohepatitis; NMCI: No Mild Cognitive Impairment; MCI: Mild Cognitive Impairment; Oral SDMT, Symbol digit modalities test (oral version). TR, Total number of characters processed; TOT, Total correctly processed; CON, Concentration performance; RA, Total right answers; O, errors of omission; C, errors of commission; O+C, Total errors. *$p<0.05$; **$p<0.01$; ***$p<0.001$; ****$p<0.0001$ NAFL (both NMCI and MCI) and NASH (both NMCI and MCI) patients compared with the controls; differences between MCI and NMCI are indicated by: $^{a}p<0.05$; $^{aa}p<0.01$; $^{aaa}p<0.001$; $^{aaaa}p<0.0001$ for NAFL patients and by $^{c}p<0.05$; $^{cc}p<0.01$; $^{ccc}p<0.001$; $^{cccc}p<0.0001$ for NASH patients.
